# Hepatocellular Carcinoma and High Grade Neuroendocrine Carcinoma: A Case Report and Review of the Literature

**DOI:** 10.4021/wjon276e

**Published:** 2011-02-26

**Authors:** El Mehdi Tazi, Ismail Essadi, Hind M'rabti, Hassan Errihani

**Affiliations:** a Department of Medical Oncology, National Institute of Oncology, Rabat, Morocco

**Keywords:** Hepatocellular carcinoma, Neuroendocrine carcinoma, Liver tumors

## Abstract

We describe a rare hepatic collision tumor composed of a hepatocellular carcinoma and a high-grade neuroendocrine carcinoma. The patient, a 68-year-old man, underwent a partial hepatectomy because of a 4.0 cm mass. The tumor had two distinctive patterns. The majority of the tumor was a high-grade neuroendocrine carcinoma with features of a small cell carcinoma that was positive for chromogranin, synaptophysin, and cytokeratin 19 and negative for hepatocellular antigen and alpha-fetoprotein (AFP). The second component was a moderately differentiated hepatocellular carcinoma that was positive for hepatocellular antigen and AFP and negative for neuroendocrine markers. The two tumors were separated by fibrous bands. In areas where they collided, there was no transition or intermingling of cells between the two components, thus, it is different from the combined type of tumors. After removal of the tumor, the patient underwent four courses of chemotherappy which included etoposide and cisplatin with a follow-up period of 28 months.

## Introduction

A collision tumor is an unusual neoplasm that is defined as having two histologically distinct tumors simultaneously involving the same organ with no transition between them. They are different from combined tumors, which are not only contiguous, but also intermingle with each other. In the liver, both types are rare, but the combined type is more frequent. It represents 2.0 to 3.6% of all primary hepatic malignancies [1, 2]. It is postulated that these combined tumors arise from stem cells that evolve into divergent differentiation [3, 4]. The most frequent combined tumor consists of hepatocellular and cholangiocarcinoma (hepatocholangiocarcinoma). Hepatic collision tumors are even rarer with an incidence of 0.1 to 1% [1, 3]. Most of the collision tumors also show a hepatocellular carcinoma accompanied by a cholangiocarcinoma. Single case reports of primary collision tumors include hepatocellular carcinoma with sarcoma [5] and the rare occurrence of hepatocellular carcinoma with neuroendocrine tumor [6], such as the one described in this article.

## Case Report

**Figure 1:**
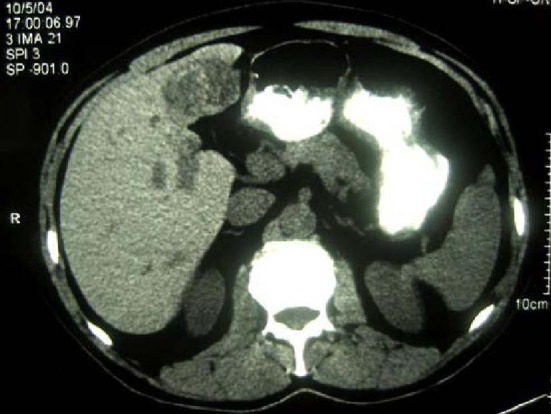
CT scan of liver showing a 4 cm mass in the left lobe with areas of necrosis.

**Figure 2:**
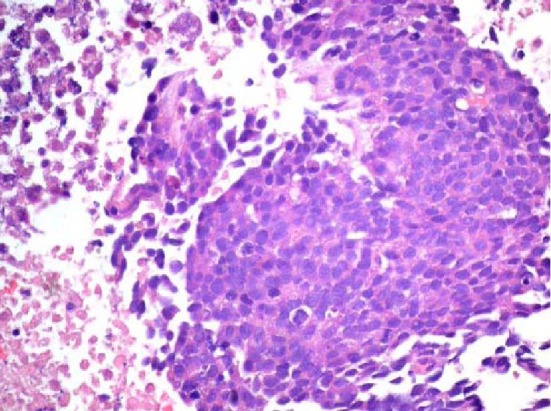
Initial liver biopsy showing a necrotic poorly differentiated carcinoma (hematoxylin and eosin, × 200).

**Figure 3:**
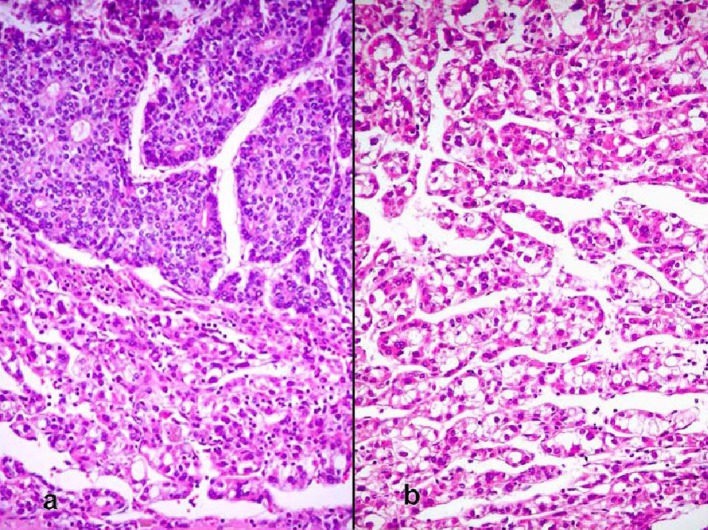
(a) Sharp demarcation of the both components of the moderately differentiated hepatocellular carcinoma and the neuroendocrine carcinoma with the latter showing rosettes (hematoxylin and eosin, × 100). (b) Hepatocellular carcinoma (hematoxylin and eosin, × 200).

**Figure 4:**
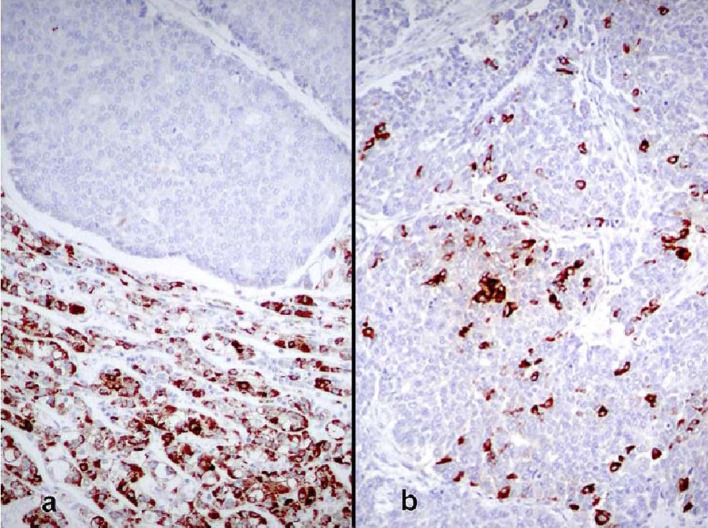
(a) Borderline area between both tumors showing immunoreactivity for Hep Par1 in the hepatocellular carcinoma, whereas it is negative in the neuroendocrine tumor (immunoperoxidase, × 100). (b) Neuroendocrine carcinoma showing immunoreactivity for chromogranin (immunoperoxidase, × 100).

The patient was a 68-year-old Hispanic man who had a medical history of hepatitis B treated with lamivudin monotherapy, which was terminated in 2001. Periodic hepatic ultrasound follow-up showed a mass on the left lobe of the liver. A CT scan of the abdomen revealed a 4.0 cm mass located in the hepatic segment IV (Fig. 1). The serum level of alpha-fetoprotein (AFP) at this time was 1,191 ng/ml. The patient had normal liver enzymes and total bilirubin of 0.4 mg/dl. CA19-9 and CA125 were normal. CT of chest and extrahepatic abdomen showed no other lesions. At our institution, a liver core biopsy was performed, which showed an extensively necrotic, epithelial malignant neoplasm (Fig. 2). An intraoperative ultrasound showed that the mass was located in segment IV extending to segment V for which an extended left hepatectomy was performed. Intraoperatively, the abdominal cavity, including omentum, peritoneum, intestines, stomach, and pancreas, were free of lesions. The specimen showed both a hepatocellular carcinoma and a neuroendocrine carcinoma (Fig. 3), confirmed by immunohistochemistry exam (Fig. 4). The patient was treated with four courses of chemotherapy regimen including Etoposide: 120 mg/m^2^ day 1 to day 3 and cisplatin: 80 mg/m^2^ day 2; the cycle was repeated every three weeks. The patient is free of recurrence with a follow-up of 28 months.

## Discussion

True primary hepatic collision tumors are unusual, but the presence of hepatocellular carcinoma growing synchronously with a neuroendocrine tumor in a patient with no existing extrahepatic tumor is even rarer. We report one such case where the hepatocellular carcinoma grew independently from an adjacent high-grade neuroendocrine carcinoma in a noncirrhotic liver. Although hepatocellular carcinoma is essentially considered a complication of liver cirrhosis, studies show that about 20 - 40% of these tumors develop in noncirrhotic livers [7, 8]. There are a few reports of primary neuroendocrine tumors in the liver combined with hepatocellular carcinoma, but these represented the differentiation of the malignant liver cells into a neuroendocrine tumor [9, 10]. The only case that appears to fulfill the criteria for collision tumor is the study reported by Ishida et al [6]. Their patient was a 72-year-old man with a 3 cm high-grade neuroendocrine carcinoma in segment 8 and a 1.5 cm moderately differentiated hepatocellular carcinoma in segment 5. The liver was cirrhotic and lymph nodes showed metastatic neuroendocrine carcinoma. However, the authors favored the view that the neuroendocrine tumor did not arise de novo and speculated that it was a hepatocellular carcinoma that underwent neuroendocrine differentiation despite the fact that it lacked morphologic, immunohistochemical, and ultrastructural features of hepatocellular carcinoma. In our case, the two tumors were independent of each other, as evidenced by their gross and microscopic features. Grossly, the tumors were separated by fibrous bands and had distinctly different color qualities. The larger tan-white and friable tumor corresponded with the morphologic features of neuroendocrine carcinoma and comprised about 70% of the mass. The green and more nodular hepatocellular carcinoma was well defined and sharply demarcated from the first tumor by fibrous bands. Microscopically, both tumors were distinctive morphologically, immunophenotypically, and ultrastructurally. They were separated by fibrous bands and they were in direct contact with each other only focally. Even in the areas of contact, the cellular components were markedly different and did not intermingle. In these areas of juxtaposition the tumors appeared to be pushing rather than infiltrating each other. Also, electron microscopy showed no neuroendocrine features in the hepatocellular carcinoma component. Immunohistochemical stains highlighted the differences between both tumors with one showing an immunophenotype of hepatocellular carcinoma while the other featured neuroendocrine markers. The rosette formation in the neuroendocrine tumor suggests that it could have been initially low or intermediate grade neoplasm that later acquired a more aggressive morphology in the form of a small cell carcinoma. These features distinguish collision tumor from the combined type reported by Barsky et al [9] and Yamaguchi et al [10] where two tumors showed intermingled cells in the transition zone that could not be morphologically separated. In addition, some of the cells in the previously reported tumors had morphologic features of hepatocellular carcinoma on paraffin sections stained by hematoxylin and eosin sections but displayed neurosecretory granules by electron microscopy. These features support the view that although morphologically different, the tumors may have originated from a liver cell that eventually acquired neuroendocrine features. Apart from the collision and combined types, neuroendocrine tumors can also occur in an isolated fashion primarily in the liver in the form of carcinoids or highgrade tumors represented by small cell carcinomas [11, 12]. However, the issue regarding the origin of primary neuroendocrine tumors of the liver is not well elucidated yet. Hepatic progenitor cells found in the epithelial lining of intrahepatic bile ducts could potentially serve as the origin of neuroendocrine tumors [13, 14]. This hypothesis is supported by the presence of carcinoids and small cell carcinomas in noncirrhotic livers. In our case, the strong positivity for CK19 in the neuroendocrine component supports the theory that these cells originate from hepatic stem cells. A second hypothesis postulates that a stem cell with pluripotential capability is the precursor for liver cell carcinoma, neuroendocrine malignancies, and other tumors with polyphenotypic expression. This is supported by descriptions of hepatocellular carcinomas with neuroendocrine features [15]. Zhao et al [16] found neuroendocrine differentiation in 60% of their hepatocellular carcinomas. This high rate is incongruent with the rarity of primary neuroendocrine tumor in the liver. The prognosis and treatment of hepatic neuroendocrine carcinoma and hepatocellular carcinoma collision tumor are uncertain due to the small number of cases studied. Likewise, it is not known which of the two components carries a negative influence on patients’ survival. The other patients with solitary high-grade neuroendocrine tumors died within a few months after diagnosis [6]. More cases of collision and combined tumors of this type need to be documented to obtain a better insight into their pathogenesis, behavior, and treatment.
